# Ideal Bridge Work

**Published:** 1894-12

**Authors:** R. A. Holliday

**Affiliations:** Atlanta, Ga.


ARTICLE III.

### IDEAL BRIDGE WORK.

BY R. A. HOLLIDAY, D. D. S., ATLANTA, GA.

It cannot be denied that some form of bridge work is the *ideal* substitute of from one to ten teeth. Of the many different bridges now in use, each claimed by its advocates to be the best, makes a selection difficult, and the case in hand must determine the kind to be used, whether removable, non removable, porcelain, all gold, or porcelain and gold.

I am aware that in advocating the non-removable bridge, much opposition will be met; but, if we consider the feelings of the patient, the satisfaction they experience in knowing that the substitute can be used without fear of its dropping or being sneezed out, and the nervous apprehension of many preventing the wearing of partial plates, the advantages of a securely anchored bridge is apparent. In the one we have a yielding, in the other a non-yielding base, allowing of perfect mastication.

A permanent or non-removable bridge, *must* have two or more points of attachment, according to the size of the bridge, and, to insure stability, the teeth or roots used should be in a healthy condition. It will not do to anchor a bridge to an unhealthy tooth or root, expecting a cure to result from such crowning. However, there is an exception, viz: where pyorrhœa has caused absorption of process and recession of gums. Such a tooth may be crowned with the assurance that it will be benefitted, and, in a majority of cases, the absorption process entirely arrested.

If the teeth used as supports are divitalized, the roots *must* be filled before the bridge is cemented on, as the cement at the time of setting the bridge cannot be depended upon to thoroughly fill the root to the apex.

Most of the failures coming under my observation can be attributed to the neglect of one of these precautions.

The teeth should rest on the alveolar ridge, and the natural contour given them—not as a saddle, which is more extensive, covering the ridge, while the *ideal*, as I conceive it, is a complete restoration, giving the same form and contour of the natural teeth. This is easily done by swagging a piece of twenty-eight guage gold, 22 to 24k., or burnishing a thin piece of pure gold or platinum on the plaster cast. The first is best, as a thick piece of gold is not as apt to pull away from the ridge when soldering. Before making a metal die the plaster model should be scraped slightly on the ridge that the bridge when completed, will allow the teeth to rest firmly against the ridge.

This form is more comfortable to the tongue, and a great deal easier to keep clean than the so-called self-cleansing spaces, which is a misnomen. It is not self-cleansing in any sense, and even in the mouths of our most careful patients extremely difficult to keep clean. It provides a place for the lodgement of food besides feeling unnatural to the tongue. I have yet to see a bridge with the self-cleansing spaces that is kept perfectly clean, and I have examined quite a number.

How often do we find plates brought to us for repairs, encrusted with tartar, exhibiting gross carelessness in caring for them. We would be safe in saying that one-half of those wearing artificial dentures fail to keep them clean, although they can remove them; how then can we expect the average patient to take proper care of a bridge? Hence, I believe that the modified saddle bridge is the very best, offering the least surface for the collection of food, coming nearest in shape, being nearest to nature in shape, being more comfortable and easiest of all to keep clean.

My experience has taught me not to rely on hollow gold dummies. I will not say that they are never perfectly soldered, but will assent that they are oftener defective; the solid dummy should be used, but in most cases I prefer the porcelain facing, as it looks neater.

An excellent and quick way of making a solid dummy, as suggested by Dr Hinman, is as follows: Select a plain rubber tooth of proper size, and press it into an asbestos block, making the depression deep enough to cast the full crown; fill this with gold scraps, melt with blow-pipe, and while in this condition press down with smooth metal surface. The result will be a perfect dummy, requiring little polish; or a mould of plaster and marble dust will give the same result.

To make a nice joint with porcelain facing, bicuspids and molars, after stamping cusps of pure gold, twenty-eight guage, paint with whiting the portion to be occupied by cusp of facing, and fill the remainder with solder. After backing facing with thin platinum, place it in cusp, which will be found to come up one-sixteenth of an inch in front of facing; attach with piece of wax until invested for soldering; remove the wax and contour with solder, being careful not to burnish the gold to buccal face of porcelain until the completion of the bridge for fear of cracking the facing. This will give as perfect a joint as can be made with gold and porcelain.

It is important to have the teeth properly contoured with solder before the final investment for uniting the bridge, as it saves time and lessens the liability of facing to crack.

In conclusion, allow me to say that bridgework has done more to stimulate the profession to greater achievement in prosthetic dentisty than anything that has occurred since Chapin A. Harris and his associates established the first dental college, while vulcanite has done more to degrade it.

Dr. D. D. Atkinson: I differ with the gentleman as regards the self-cleansing spaces. We know that some patients will not keep anything clean—a vulcanite plate, a gold plate or their own mouths—and I do not see how they can keep one clean that covers a considerable portion of the gum tissue with no space left for brushing. The fault often lies with the patient and noc with the operator or his work.

Dr. T. P. Hinman: The point used by Dr. Holliday is this: You have to heat your porcelain filling twice; but we all know that if the facings do not break in the first they are apt not to break in the second. If you simply back them up with platinum or gold and flow your solder clear around, do you not see that the contraction of the solder, or consolidation, will draw these faces together, and the lateral pressure may crack them? On the contrary, if we back up each tooth separately, finishing it and having it all ready, then all you have to do is to solder the little joints together. The idea is to restore to the mouth what nature had there, and the part of the gum that is covered by the dummy is just exactly as much as was covered by the original tooth, it resting lightly against the gum. I will guarantee nothing can get under it. It is the cleanest bridge work I know of.

Dr. H. H. Johnson: Where there is a self-cleansing space you have no irritation, and accumulations can be brushed out as easily as brushing the natural teeth. Anything pressing on the gum produces irritation. It seems to me almost impossible to press anything covering a considerable area into the gum sufficiently hard to prevent food and other accumulations getting under it and keeping it there permanently without producing considerable inflammation. I contend that a bridge properly constructed, with self-cleansing spaces and smoothly finished, can be kept clean as easily as any natural teeth. There are very few sets of natural teeth that are kept perfectly clean and free from odor. A silk thread will detect odor in almost any set of teeth.

Dr. W. G Browne: I presume by dummies the essayist refers to attempting to make hollow gold crowns. I want to say that can be obviated in two ways without making a solid gold crown. One way is to make a dummy and fill it with cement, soldering on the other end afterwards. The other is to make the bridge and vulcanize rubber into and filling the dummy crowns. The use of vulcanized rubber is occasionally useful in places where there is a great length of bridge to be made. It answers every purpose and is more easily and cheaply done.

Dr. J. H. Thompson: I agree with Dr Johnson as to the construction of bridges. I know several bridge workers have adopted that method of saddle and allowing the bridge to rest on the gum, claiming that it is cleaner than the others. They say *that* because they do not see under it. But the filth is there just the same.

I cannot agree with Dr. Browne on the rubber question. As to filling the crown with cement, I doubt if it can be hermetically sealed. The dummies should be filled solid with solder.

Dr. Browne: Teeth *can* be filled with cement and hermetically sealed. I say that a dentist who has done this at various times is better authority than the man who thinks it cannot be done.

Dr. J. M. Mason: Have you ever examined one of those crowns after it went through the heat?

Dr Browne: Yes, sir; and it gets harder and harder the more you heat it.

Dr. H. R. Jewett: I want to call attention to a new process of bridge-work—the Hollingsworth. It is regarded by those who have used it as the best method. It aims at making a complete and solid piece of work. This process stamps up the entire coronal together; it stamps up the entire facings together, and it solders the dummies in one solid piece of material. The ideal bridge is a perfectly solid piece, free from bubbles and air holes, which you often get when attempting to fill and solder dummies together.—*Southern Dental Journal.*

